# Electrophysiological Evidence in Schizophrenia in Relation to Treatment Response

**DOI:** 10.3389/fpsyt.2018.00259

**Published:** 2018-06-13

**Authors:** Kazuki Sueyoshi, Tomiki Sumiyoshi

**Affiliations:** Department of Preventive Intervention for Psychiatric Disorders, National Institute of Mental Health, National Center of Neurology and Psychiatry, Kodaira, Japan

**Keywords:** electroencephalogram, event related potentials, LORETA, cognition, schizophrenia

## Abstract

Several domains of cognitive function, e.g., verbal memory, information processing, fluency, attention, and executive function are impaired in patients with schizophrenia. Cognitive impairments in schizophrenia have attracted interests as a treatment target, because they are considered to greatly affect functional outcome. Electrophysiological markers, including electroencephalogram (EEG), particularly, event-related potentials, have contributed to psychiatric research and clinical practice. In this review, we provide a summary of studies relating electrophysiological findings to cognitive performance in schizophrenia. Electrophysiological indices may provide an objective marker of cognitive processes, contributing to the development of effective interventions to improve cognitive and social outcomes. Further efforts to understand biological mechanisms of cognitive disturbances, and develop effective therapeutics are warranted.

## Introduction

Cognitive impairments are considered as a fundamental feature of schizophrenia (1). Patients with the illness present disturbances across several cognitive domains, such as executive function, some types of memory, attention, fluency, and information processing/speed (2, 3). Cognitive function predicts social function more accurately than psychotic symptoms, and has been drawing attention as target of treatment (4, 5).

The Measurement and Treatment Research to Improve Cognition in Schizophrenia (MATRICS) Consensus Cognitive Battery (MCCB) (6) and the Brief Assessment of Cognition in Schizophrenia (BACS) (7) have been developed to evaluate disturbances of cognitive function in schizophrenia. Also, as an interview-based multidimensional assessment tool of social function, the Specific Level of Functioning Scale (SLOF) has been implemented (8). In fact, social functioning, as measured by the SLOF, has been shown to be correlated with cognitive function, as measured by the BACS in patients with schizophrenia (9).

There is evidence for the role of electrophysiological measures as an objective marker of neuropsychological performance (10–13). In fact, electrophysiological responses generally precede behavior-based cognitive performances, and are also useful to predict treatment outcome regarding cognitive disturbances (10, 14, 15). This paper provides selective reviews of studies on the relationships among cognitive function, electrophysiological findings, and treatment response in patients with schizophrenia.

## Electrophysiological evidence in schizophrenia

### Spontaneous electroencephalogram (EEG)

In general, functional neuroimaging techniques measuring blood flow and metabolism, such as functional magnetic resonance imaging (fMRI), positron emission tomography (PET), and Single photon emission computed tomography (SPECT) may not directly differentiate between activation and inhibition of a specific brain region (16). On the other hand, EEG consists of components of electrical activities that are inhibitory (e.g., slow “delta” frequencies), excitatory (e.g., fast “beta” frequencies) or steady-state (mid-range “theta” and “alpha” frequencies) in nature (16). Also, EEG has an advantage in terms of time resolution compared to other techniques to evaluate brain functions.

Imaging of electrophysiological activity, such as EEG, is feasible and cost-effective. For example, Pascual-Marqui et al. developed the low-resolution brain electromagnetic tomography (LORETA) (16), which is a source localization analytic estimator. The purpose of current source localization is to overcome the volume conductance problem in EEG analyses and cope with the reference confounding effects (16). Neuroleptic-naïve patients with first-episode schizophrenia have been reported to demonstrate hyperactivity of delta band in the frontal-prefrontal area and hypoactivity of middle range band (theta and alpha) in the left temporal parietal area by means of LORETA (16). These findings support the concept that cognitive disturbances of schizophrenia are generated by inhibition of frontal and left temporal areas (17).

Functional deviations of frontal lobes are reflected by disturbances of executive function and working memory in schizophrenia (18, 19). In fact, a meta-analysis of studies using fMRI and PET reports reduced activation in dorsolateral prefrontal cortex and anterior cingulate cortex during executive functioning task performance in patients with schizophrenia (18). The dysfunction related to auditory verbal hallucinations (20) is consistent with the role for the left temporal lobe in auditory perception and language processing (21, 22).

Inhibited activities of the left temporal area in schizophrenia are also demonstrated by using PET (23). Further, dysfunction of fronto-temporal connectivity has been reported in schizophrenia (24), consistent with Fletcher et al. suggesting the role for this anatomical complex in the psychopathology of schizophrenia (17). Accordingly, an fMRI study reported the relation of fronto-temporal connectivity with cognitive functions, including working memory (25). The reduction of blood flow and metabolism in the frontal and left temporal areas in schizophrenia was supported by Pascual-Marqui et al. (16) who found inhibition of electrical activities in these brain regions.

On the other hand, there is a report that mid-fast band frequencies were not altered in medicated-free patients with schizophrenia (26), although delta band activities were increased. In this line, an increase in the delta activity was noted in frontal areas, left inferior temporal gyrus, and parahippocampal gyrus of neuroleptic-naïve patients with schizophrenia, as revealed by LORETA (27).

### Event-related potentials

Event-related potentials (ERPs) are linked in time with physical and mental events, and are typically extracted from the scalp-recorded EEG by means of signal averaging (28). ERP components, such as P50, mismatch negativity (MMN), and P300, provide neural activities associated with sensory-perceptual and cognitive events in the order of milli-seconds (29). P50 and MMN reflects attention-independent (pre-attentive) automatic information processes, while P300 has been used as a measure of attentive information processes (30).

P50 is a pre-attentional component recorded about 50 ms after the presentation of an auditory stimulus in the conditioning-testing paradigm. Its amplitude is suppressed when a second click sound is presented 500 ms after an initial click (31). The P50 suppression is thought to reflect a sensory gating mechanism aimed at protecting against information overload (32). A meta-analysis study has reported robust P50 suppression deficits in schizophrenia (33). Specifically, deficits of P50 suppression have been linked to poor performance on tests of cognitive domains, such as attention (34–36), working memory (11, 36), processing speed (11, 34), and executive function (35). These associations suggest that impaired P50 sensory gating provides a targets of interventions to alleviate cognitive disturbances of schizophrenia (11).

MMN is typically recorded in the condition where a subject is instructed to divert attention from stimuli generated by the auditory oddball task (37). MMN is generated when a stimulus violates the invariance or regularity of the recent auditory past. For example, MMN is recorded when an deviant stimulus that differs in frequency, duration, intensity, or location is presented among repeatedly presented standard stimuli (38). MMN is considered to provide an index of (1) auditory sensory or echoic memory, and (2) context-dependent information processing at the level of the primary and secondary auditory cortices (38). Parameters of MMN, e.g., amplitudes and latencies, are thought to reflect the first step in a chain of events leading to the conscious detection of differences between auditory stimuli and variance in the auditory environment (38).

Reduction of MMN amplitudes in patients with schizophrenia shows a large effect size as demonstrated by meta-analysis (38). Specifically, patients with chronic schizophrenia show a decrease in MMN current density in the right medial frontal gyrus, right cingulate gyrus, and right paracentral lobule (39). Altered MMN amplitudes have been associated with impairment of cognitive functions, such as attention (12, 40, 41), processing speed (41, 42), verbal learning (40, 43), verbal fluency (44), and executive function (42). Also, its amplitudes have been linked to functional outcomes (45–47). Overall, pre-attentive information processes serve as a gateway to higher cognitive and psychosocial functioning (12). Further, the ability of MMN to reflect functional outcomes have been reported to be better than those of behavior-based cognitive performances and social cognition (15). These considerations further support the utility of MMN as a marker of treatment effects on social functioning.

P300 is typically recorded when a subject is required to pay attention to infrequent stimuli in an auditory oddball task (48). Amplitudes of P300 waveforms, thought to reflect cognitive processes such as directed attention and the contextual updating of working memory (31), are reduced, and the latency of P300 are delayed in patient with schizophrenia (33). Altered P300 activities have been reported to correlate with clinical symptoms of schizophrenia (37). By means of LORETA, current sources of P300 were estimated to reside in the bilateral medial frontal and medial parietal cortex, bilateral superior temporal gyrus, right temporo-parietal junction, and left lateral prefrontal cortex (37).

P300 amplitudes have been shown to positively correlate with performance on tests of verbal learning (49), organization and discriminability of memory (13), attention (50), verbal fluency (49), and executive function (49). Also, prolonged latency of P300 has been associated with performance on tests of verbal learning (13) and verbal fluency (51). It is important that these domains of cognition are related with functional capacity and real-world functions (9, 52). Further, a correlation has been reported between P300 amplitudes and functional capacity (53). These considerations support the potential utility of P300 as a biomarker to predict treatment response (53).

## Electrophysiological changes during treatment

### Spontaneous EEG

Using above-mentioned electrophysiological markers, some studies have reported the effect of treatment on cognitive disturbances of schizophrenia. Repetitive transcranial magnetic stimulation produced the following changes in patients with schizophrenia (54); (1) an increase in delta band activities in bilateral anterior cingulate gyrus, (2) a decrease in beta-1 and beta-3 band in the middle temporal lobe ipsilateral to the site of stimulation, and (3) an increase in beta-2 band in the middle temporal and inferior parietal lobule on the right side. In the same study (54), brain metabolism using ^18^FDG-PET was simultaneously measured. While the change of current density of beta bands activities was in accordance with the PET findings, that of delta band was not correlated with brain metabolism (54).

### ERPs

Using traditional ERP methods, some authors have investigated the effect of atypical antipsychotic drugs on cognitive function in schizophrenia. As to P50 suppression, treatment with quetiapine of antipsychotic-naïve first-episode patients improved the sensory gating deficits (55). In addition, some atypical antipsychotics, such as clozapine (56, 57) and risperidone (58), showed efficacy for the recovery of P50 suppression.

In treatment studies for the deficits of MMN in schizophrenia, aripiprazole has been reported to increase MMN amplitudes (59). On the other hand, other atypical antipsychotic drugs, such as clozapine (60), risperidone (61), and olanzapine (62) have been shown not to affect MMN amplitudes. Further study on the ability of medication to alleviate altered MMN parameters in the illness is warranted.

In the P300 study, a controlled double-blind trial investigated the effect of clozapine or haloperidol on ERPs, including P300 and MMN, in chronic schizophrenia (60). Treatment with clozapine, but not haloperidol was associated with an increase in P300 amplitudes (60). In another study, clozapine similarly increased P300 amplitudes, and also enhanced performance on working memory tasks (63). On the other hand, the effect of olanzapine on P300 has not been consistent (62, 64–66). Perospirone did not significantly affect P300 in schizophrenia (67).

Using three dimensional images of current density of ERPs in the brain, we reported the ability of treatment with olanzapine for 6 months to enhance P300 current density in the left STG, yielding a distribution pattern of the current density similar to that in healthy control subjects (68). A later study confirmed treatment with olanzapine was associated with increase of P300 current source density in the left STG (69). Importantly, the degree of increase of P300 in the left STG was positively correlated with improvement in negative symptoms and verbal learning memory, while improvement of quality of life was associated with an increase of P300 in the left prefrontal cortex (69). On the other hand, treatment with perospirone was found to improve P300 current density in the left prefrontal cortex, which was related with improvement of daily-living skills, as measured by the script task (70). These findings suggest LORETA imaging of P300 is a useful indicator of treatment response in some aspects of the psychopathology and functional outcomes of schizophrenia.

## Clinical implications

Early intervention into schizophrenia and related conditions has been suggested to improve the prognosis of patients. Accordingly, shorter duration of untreated psychosis has been associated with better long term outcomes (71). Electrophysiological measures may be useful to evaluate the risk for developing psychosis. For example, P300 amplitudes are reduced in the prodromal stage (72, 73). Specifically, treatment with perospirone in an ultra-high risk case immediately before the onset of schizophrenia was shown to “normalize” cognitive function and social outcomes 3 years later. Importantly these neuropsychological and clinical events were preceded by improvement of P300 amplitudes (14). Also, MMN amplitudes have been shown to identify high-risk individuals who later develop overt schizophrenia (44, 74). Taken together, electrophysiological indices may provide a sensitive marker to evaluate treatment effects, including those related to cognitive function, and in some cases, predict the risk of psychosis.

## Conclusions

In this review, we have provided a summary of studies relating electrophysiological findings to cognitive performance in schizophrenia. Electrophysiological indices may provide an objective marker of cognitive processes, contributing to the development of effective treatment of cognitive and social outcomes. Further efforts to understand electrophysiological mechanisms of cognitive disturbances, and develop effective therapeutics are warranted.

## Author contributions

All authors listed have made a substantial, direct and intellectual contribution to the work, and approved it for publication.

### Conflict of interest statement

The authors declare that the research was conducted in the absence of any commercial or financial relationships that could be construed as a potential conflict of interest.
